# Structural Elucidation of Alkali Degradation Impurities of Favipiravir from the Oral Suspension: UPLC-TQ-ESI-MS/MS and NMR

**DOI:** 10.3390/molecules27175606

**Published:** 2022-08-31

**Authors:** Ravi Patel, Abhishek Dube, Ravisinh Solanki, Dignesh Khunt, Shalin Parikh, Vijayabhaskarreddy Junnuthula, Sathish Dyawanapelly

**Affiliations:** 1 Graduate School of Pharmacy, Gujarat Technological University, Gandhinagar 382028, Gujarat, India; 2 Senores Pharmaceuticals Pvt. Ltd., Ahmedabad 380058, Gujarat, India; 3 Drug Research Program, Faculty of Pharmacy, University of Helsinki, Viikinkaari 5 E, 00790 Helsinki, Finland; 4 Department of Pharmaceutical Sciences and Technology, Institute of Chemical Technology, Mumbai 400019, Maharashtra, India

**Keywords:** RP-HPLC, stability indicating, COVID-19, UPLC-MS/MS, characterization, favipiravir, degradation products

## Abstract

A novel stability-indicating, reversed-phase, high-performance liquid chromatography (RP-HPLC) method was developed and validated for the determination of favipiravir in an oral suspension. The effective separation of favipiravir and its degradation products was achieved on a Zorbax Eclipse Plus C18 column (5 μm particle size, 150 mm length × 4.6 mm diameter). The mobile phase was prepared by mixing 5 mM of phosphate buffer (pH 3.5) and methanol in a 75:25 *v/v* ratio delivered at a 1.0 mL/min flow rate. The eluents were monitored using a photodiode array detector at a wavelength of 322 nm. The stability-indicating nature of this method was evaluated by performing force degradation studies under various stress conditions, such as acidic, alkali, oxidative, thermal, and photolytic degradation. Significant degradation was observed during the alkali stress degradation condition. The degradation products generated during various stress conditions were well separated from the favipiravir peak. In addition, the major degradation product formed under alkali stress conditions was identified using UPLC-ESI-TQ-MS/MS and NMR. Method validation was performed according to the ICH Q2 (R1) guideline requirements. The developed method is simple, accurate, robust, and reliable for routine quality control analysis of favipiravir oral suspensions.

## 1. Introduction

**Figure 1:**
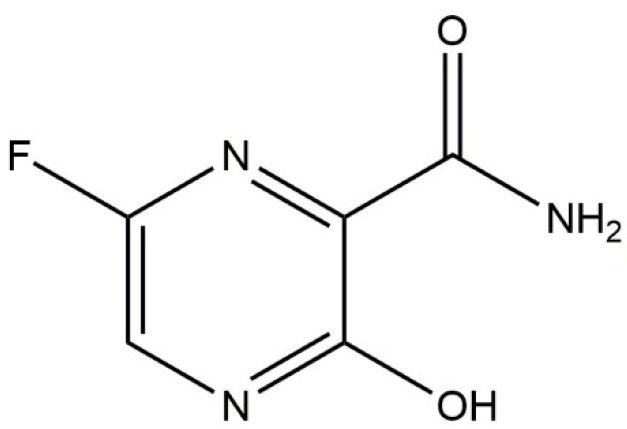
Structure of favipiravir.

COVID-19 is a global pandemic, and it was first discovered in Wuhan, China [1]. To date, a variety of treatments have been established; however, antiviral agents are prominent. Favipiravir, 6-fluoro-3-hydroxypyrazine-2-carboxamide (Figure 1), is an oral antiviral drug [2] and has been recently approved for the management of novel COVID-19 in many countries [3,4]. Favipiravir inhibits RNA polymerase, which is required for viral replication, in a targeted manner. Favipiravir has also been studied to treat life-threatening infections such as Ebola, Lassa, and, most recently, COVID-19 [5,6]. In humans, the percentage of favipiravir binding to plasma protein was reported to be 54% [7]. Various formulation approaches in the literature are trying to improve the bioavailability of poorly soluble drugs for various indications [8,9,10,11,12,13,14,15,16,17,18].

Favipiravir tablets available on the market for antiviral indications have 800 mg of label claim. For COVID-19 treatment, the recommended dose of favipiravir is approximately 1800 mg. Hence, patients need to take multiple tablets at a time during treatment. The Indian regulatory agency has approved a favipiravir oral suspension of 100 mg/mL to overcome multiple tablets [19].

Different chromatographic methods for quantifying favipiravir, including favipiravir analysis in human plasma by LC-MS/MS and HPLC, have been reported [20,21,22,23,24,25,26]. The HPLC method for the determination of favipiravir in bulk and the characterization of degradation products was also reported [27]. Currently available methods of favipiravir consist of spectrophotometric and spectrofluorimetric determinations in the dosage form other than oral suspension [27]. However, no method has addressed the issue of stability, indicating that the method for favipiravir is the most unstable dosage form of oral suspensions.

The present research work aimed to develop a stability-indicating RP-HPLC method for the determination of favipiravir from an oral suspension. Characterization of the alkali degradation product was performed using UPLC-TQ-ESI-MS/MS and ^1^H NMR spectroscopy.

## 2. Materials and Methods

### 2.1. Chemicals and Reagents

The favipiravir working standard (potency: 99.97%) was provided by BDR Pharmaceutical International Pvt. Ltd. (Vadodara, Gujarat, India). The favipiravir oral suspension marketed by FDC Limited (Mumbai, India) used in this study was procured from a pharmacy. Methanol and acetonitrile were procured from Merck (Darmstadt, Germany). Formic acid, ortho-phosphoric acid, potassium dihydrogen phosphate (HPLC grade), hydrochloric acid, 30% hydrogen peroxide, and sodium hydroxide (AR grade) were procured from Sigma-Aldrich (St. Louis, MO, USA). Water was HPLC grade (Millipore Milli-Q-system). A mixture of water and methanol at a ratio of 60:40 *v*/*v* was used as a diluent.

### 2.2. Instruments

An Agilent 1260 Infinity II series liquid chromatography system equipped with a quaternary pump (G7118B) was connected to an autosampler (G7129A). A Zorbex Eclipse plus C18 (150 mm × 4.6 mm, Agilent 5 µm) column was used as a stationary phase for the chromatographic separation. All the data were processed using Agilent open Lab CDS software. A weighing balance (Shimadzu, Kyoto, Japan), an ultra sonicator, a mobile phase filtration assembly (Borosil 5350024), a centrifuge (CPR-24 Plus, Remi, Mumbai, India), an AvanceCore 400 MHz NMR spectrometer (Bruker), and a 0.22 µm membrane filter (Millipore) were used for method development, validation, and characterization studies.

### 2.3. Chromatographic Conditions

#### 2.3.1. RP-HPLC Conditions

The mobile phase was comprised 5 mM of potassium di-hydrogen orthophosphate monohydrate (KH_2_PO_4_. H_2_O) (pH 3.5 adjusted with ortho-phosphoric acid) mixed with methanol at a ratio of 75:25 *v/v*. Furthermore, the mobile phase was filtered using a 0.22 µm membrane filter and pumped through an Agilent Zorbax Eclipse plus C18 column (150 mm × 4.6 mm, 5 μm) at a flow rate of 1.0 mL/min at 25 °C. A 10 µL volume of sample was injected and monitored at a 322 nm wavelength.

#### 2.3.2. UPLC-ESI-TQ-MS/MS Conditions

An Agilent-6470 triple quadrupole (LC/TQ) mass spectrometer equipped with an electrospray ionization source was used for mass measurements and MS/MS studies of the alkali degradation product of favipiravir. To make the mobile phase LC-MS compatible, the phosphate buffer was replaced with 0.1% formic acid in water without changing the other chromatographic conditions. Ultra-pure nitrogen was used as a nebulizer and curtain gas. Collision-induced dissociation was achieved with nitrogen as the collision gas. The MS/MS conditions were set as follows: 300 °C drying gas temperature; 7 L/min drying gas flow; 25 psi nebulizer pressure; 3000 V capillary voltage (negative); 350 °C vaporizer temperature; and 200 ms dwell time. The data were collected over the m/z range from 10 to 1000. Interpretation of the data was performed by the mass-hunter software.

### 2.4. Preparation of Analytical Solution

#### 2.4.1. Standard Preparations

A stock solution of favipiravir (1000 µg/mL) was prepared by dissolving the appropriate amount of favipiravir working standard in the diluent. A working solution of favipiravir (100 µg/mL) was prepared by adequately diluting the stock solution with diluent.

#### 2.4.2. Sample Preparation

The favipiravir oral suspension (equivalent to 100 mg of favipiravir) was transferred to a 100 mL volumetric flask, added to the appropriate amount of diluent, sonicated for 30 min, and then brought up with the diluent. The samples were centrifuged at 5000 rpm to obtain a clear supernatant. Then, 1 mL of this clear supernatant was pipetted out and diluted to 10 mL with diluent.

### 2.5. Method Validation

#### 2.5.1. System Suitability

Five replicate injections of working solution of favipiravir were injected into the RP-HPLC to check the system suitability. The system suitability test was passed if the relative standard deviation, tailing factor, and theoretical plates for the favipiravir peak were NMT 2.0%, <2.0, and ≥2000, respectively [28].

#### 2.5.2. Specificity

Specificity was checked by performing force degradations of the favipiravir oral suspension under various stress conditions, such as acid, alkali, peroxide, thermal, and photolytic conditions. The intensity of stress conditions was optimized to achieve significant degradation. Degradation samples were analyzed by the RP-HPLC-PDA for the determination of assay and peak purity of favipiravir. UPLC-ESI-TQ-MS/MS analyses were carried out for structure elucidation and identification of the alkali degradation product [28].

##### Acid Degradation

An acid degradation sample of the favipiravir oral suspension was prepared by transferring an oral suspension equivalent to 100 mg of favipiravir to a 100 mL volumetric flask, adding the appropriate amount of diluent, and sonicating for 30 min. Acid hydrolysis of the sample was carried out by adding 1 mL of different normal solutions of hydrochloric acid (1 N/2 N/5 N HCl) into the flask. The sample was heated in a water bath at 80 °C for 60 min to increase the rate of degradation. The flask was cooled at room temperature and neutralized with 1 mL of the respective normal solution of sodium hydroxide, and then the volume was restored using diluent. The samples were centrifuged at 5000 rpm to obtain a clear supernatant. Then, 1 mL of this clear supernatant was pipetted out and diluted to 10 mL with diluent. This solution was injected into the RP-HPLC-PDA system.

##### Alkali Degradation

An alkali degradation sample of the favipiravir oral suspension was prepared by transferring an oral suspension equivalent to 100 mg of favipiravir to a 100 mL volumetric flask, adding an appropriate amount of diluent, and sonicating for 30 min. Alkali hydrolysis of the sample was carried out by adding 1 mL of different normal solutions of sodium hydroxide (1 N/2 N/5 N NaOH) into the flask. The sample was heated in a water bath at 80 °C for 60 min to increase the rate of degradation. The flask was cooled at room temperature and neutralized with 1 mL of the respective normal solution of hydrochloric acid, and then the volume was restored using diluent. The samples were centrifuged at 5000 rpm to obtain a clear supernatant. Then, 1 mL of this clear supernatant was pipetted out and diluted to 10 mL with diluent. This solution was injected into the RP-HPLC-PDA system.

^1^H NMR spectra were acquired at an ^1^H frequency of 399.871 MHz using an AvanceCore 400 MHz NMR spectrometer (Bruker).

##### Oxidative Degradation

A peroxide degradation sample of the favipiravir oral suspension was prepared by transferring an oral suspension equivalent to 100 mg of favipiravir to a 100 mL volumetric flask, adding an appropriate amount of diluent, and sonicating for 30 min. Oxidative degradation of the sample was carried out by adding 1 mL of different strength solutions of hydrogen peroxide (10% and 30% H_2_O_2_) into the flask. The sample was heated in a water bath at 80 °C for 60 min to increase the rate of degradation. The flask was cooled at room temperature, and the volume was restored using diluent. The sample was centrifuged to obtain a clear supernatant. Then, 1 mL of this clear supernatant was pipetted out and diluted to 10 mL with diluent. This solution was injected into the RP-HPLC-PDA system.

##### Thermal Degradation

A thermal degradation sample of the favipiravir oral suspension was prepared by transferring an oral suspension equivalent to 100 mg of favipiravir to a 100 mL volumetric flask, adding the appropriate amount of diluent, and sonicating for 30 min. The flask was placed in a water bath at 80 °C for 2 h for thermal degradation. The flask was cooled at room temperature, and the volume was restored using diluent. The sample was centrifuged to obtain a clear supernatant. Then, 1 mL of this clear supernatant was pipetted out and diluted to 10 mL with diluent. This solution was injected into the RP-HPLC-PDA system.

##### Photolytic Degradation

A photolytic degradation sample of the favipiravir oral suspension was prepared by transferring a previously exposed oral suspension (UV chamber at 254 nm, 24 h) equivalent to 100 mg of favipiravir, adding an appropriate amount of diluent, and sonicating for 30 min, and the volume was restored using diluent. The sample was centrifuged to obtain a clear supernatant. Furthermore, 1 mL of this clear supernatant was pipetted out and diluted to 10 mL with diluent. This solution was injected into the RP-HPLC-PDA system.

#### 2.5.3. Linearity

A series of standard preparations were prepared for FAV over the range of 50% to 150% of the target concentration (100 µg/mL). The linearity of the response for favipiravir was determined by preparing and injecting solutions in triplicate at every five levels. Linear regression analysis was used to determine the linear response of favipiravir, and the correlation coefficient was calculated (acceptance criteria: r2 ≥ 0.998).

#### 2.5.4. Accuracy

The pre-analyzed sample was spiked with known quantities of favipiravir at three distinct levels in triplicate for accurate investigation. Accuracy was tested at levels 1, 2, and 3 (50, 100, and 150%) of the sample concentration. The amount of favipiravir retrieved from the samples was determined using the proposed approach (98–102% acceptability criteria).

#### 2.5.5. Precision

System precision, method precision, and intermediate precision have all been studied in precision studies. The precision of the system was tested by analyzing a standard solution five times. The favipiravir peak area count (RSD) was computed. To determine the method’s level of precision, six different preparations of favipiravir oral suspension were tested against the favipiravir standard solution.

On a different day, the intermediate precision experiment was conducted with a different lot of columns. Between the two datasets, the overall RSD for the assay was computed.

#### 2.5.6. Limit of Detection and Quantification

The method’s LOD and LOQ were tested by first creating a series of standard samples with varying concentrations of favipiravir. These samples were then injected into an HPLC system. The concentrations of the favipiravir standards were 5 µg/mL, 2.5 µg/mL, 1.25 µg/mL, 0.62 µg/mL, 0.32 µg/mL, and 0.15 µg/mL.

#### 2.5.7. Robustness

To test the versatility of the method, we varied many parameters, including the wavelength of detection (5 nm), column oven temperature (5 °C), composition of the mobile phase (2% absolute), flow rate (10%), and pH of the buffer (0.2 units). The suitability of the standard solution for each variable situation was evaluated. The theoretical plates, tailing factor, and percent RSD area of the standard solution were computed for each set of data. The oral suspension sample solution of favipiravir was made in triplicate and tested under each condition to determine the percent LC (label claim) of favipiravir. Overall %RSD between the method precision and the variable condition data revealed method robustness (acceptance criterion: overall %RSD of Assay ≤ 2.0).

#### 2.5.8. Solution Stability

Standard and sample favipiravir oral suspension solutions were maintained at 25 °C. Both solutions were studied immediately and at different periods. The percentage difference between the mean initial area count and the deviation was calculated (acceptance criteria: ±2.0%).

## 3. Results and Discussion

### 3.1. Method Development and Optimization

**Figure 2:**
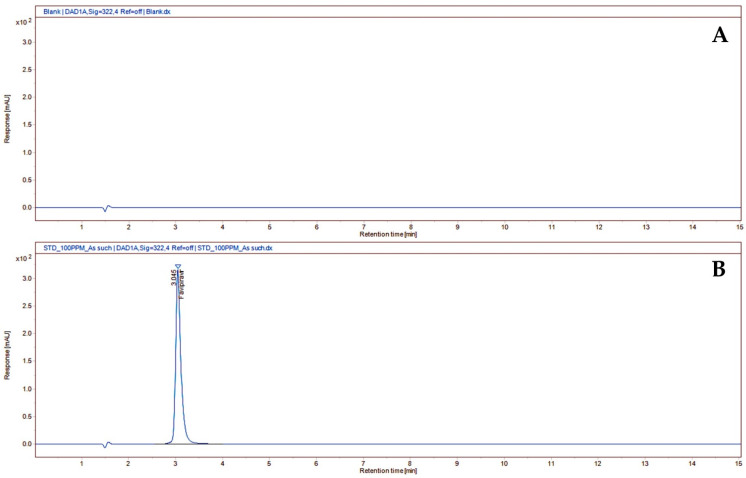
Typical HPLC chromatogram of the blank (**A**) and standard of favipiravir (**B**).

Method development for favipiravir estimation from the oral suspension was aimed at creating a method that would allow for the separation of the drug itself from its degradation products. Oral suspension formulations are less stable than tablets or capsules at recommended storage temperatures (below 30 °C) [29]. Hence, the developed assay method can resolve favipiravir from its degradants and can be used to estimate its content in stability and aged samples. Moreover, this has been supported by the degradation of favipiravir under different stress conditions. As a result, the degradants that may be generated during stability can be distinguished using the proposed test method of oral suspensions. Variables such as the mobile phase composition and buffer pH were tinkered with to improve the chromatographic method. To separate favipiravir from its degradation products, the composition and ratio of the mobile phase were crucial. We tried several different mobile phases, but the one that produced a good separation and a symmetrical peak was a 75:25 *v/v* mixture of 5 mM of phosphate buffer with a pH of 3.5 and methanol using a Zorbax eclipse plus C18 column (150 × 4.6 mm, 5 µm) with only 15 min of run time. The cost-effectiveness of the method can be seen in the selection of columns and the shorter run time. Here is an example chromatogram with a flow rate of 1 mL/min obtained with the aforementioned mobile phase and 10 µL of assay preparation injected. Approximately 3 min was discovered to be the favipiravir retention time. At the peak retention time of the favipiravir peak, there was no interference from the blank (Figure 2).

### 3.2. Method Validation

The established RP-HPLC method was validated according to ICH Q2 (R1) guidelines [30,31].

#### 3.2.1. System Suitability

During the validation study, the standard solutions were injected as a part of the system suitability test. The theoretical plates and tailing factor for the favipiravir peak were calculated using the Open Lab CDS software. For the five injections, the percentage of RSD was calculated. There was a tailing factor of 1.31, the theoretical plates were 3437.76, and the percent RSD calculated for the favipiravir peak from the five replicate injections of standard preparations was 0.29 percent. Overall, we were able to meet or exceed all three of these system suitability parameters.

#### 3.2.2. Degradation Behavior of Favipiravir in an Oral Suspension

**Figure 3:**
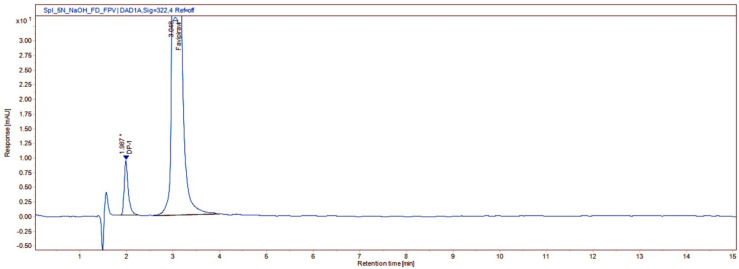
Chromatogram of alkali degradation under 5 N NaOH of favipiravir in its oral suspension.

**Figure 4:**
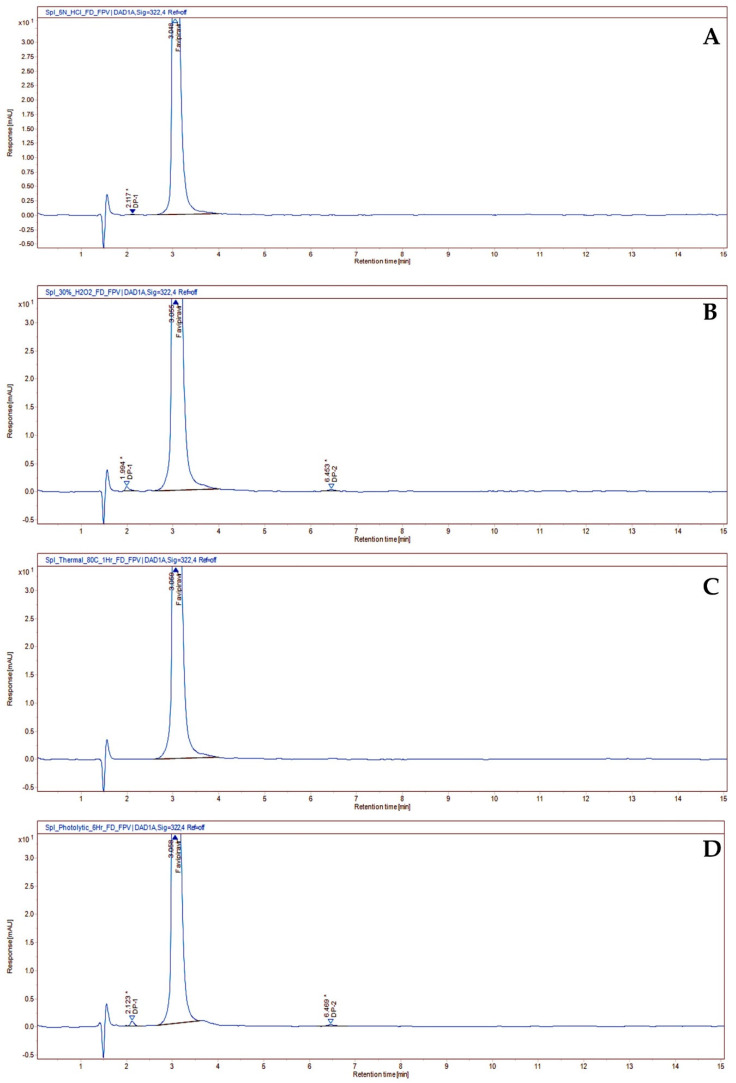
Chromatogram of acid degradation under 5 N HCl (**A**); peroxide degradation under 30%H_2_O_2_ (**B**); thermal degradation under 80 °C (**C**); and photocatalytic degradation under 6 h UV light (**D**) of favipiravir in its oral suspension.

Degradation of favipiravir was not observed under 1 N HCl, 2 N HCl, 5 N HCl (acid hydrolysis) (Supplementary Figure S1), 1 N NaOH (alkali hydrolysis) (Supplementary Figure S2), 10% H_2_O_2_ (oxidation) (Supplementary Figures S3 and S4), thermal (60 °C 2 h and 80 °C 1 h), and photolytic conditions (Supplementary Figures S5 and S6). Significant degradation of favipiravir was observed in 5 N NaOH (alkali hydrolysis: 28.50% degradation). One degradation product (DP1) was formed under alkaline conditions (Figure 3). In addition, oxidative and photolytic processes generated two more degradation products (DP1 and DP2) (Figure 4). PDA peak purity test results confirmed that the favipiravir peak was uniform and pure in all stress samples. The assay, total impurities, and peak purity of the favipiravir-forced degradation studies are given as supportive evidence (Table 1). As a result, favipiravir assays were unaffected by favipiravir’s potential impurities, confirming their specificity, selectivity, and stability-indicating power.

#### 3.2.3. Linearity

The linearity of the method can be demonstrated by validating it over the standard concentration range of 49.80 µg/mL to 149.50 µg/mL. The peak response versus concentration data yielded Y = 23.929x + 4.14, and the correlation coefficient was 0.9993, which indicates that the response is linear over the defined range.

#### 3.2.4. Accuracy

Three different sample preparations were examined, and the recovery rates ranged from 99.2% to 100%. The method is precise, as evidenced by the inclusion of percent recovery in the acceptance criteria as well as the individual and overall percent RSD of percent recovery. Table 2 summarizes the findings.

#### 3.2.5. Precision

As the acceptance criterion for the individual and overall percent RSD should not be greater than 2.0 for precision, the HPLC system has an acceptable level of precision. Because the percent RSD of the area counts was only 0.29 after five injections of the standard solution, the system appears to be accurate. As the percent RSD of favipiravir in the sample was 0.79, the method is also accurate. The percent RSD of favipiravir was found to be 0.79 for Set I (Method precision data) and 0.93 for Set II, which were performed on different HPLC systems and columns of different lots (Supplementary Figure S7)), and the overall percent RSD value was 0.86, so the method is robust. Table 3 summarizes the findings.

#### 3.2.6. Limit of Detection and Quantification (LOD and LOQ)

To determine LOD and LOQ, the signal to noise ratios (S/N) of 3:1 and 10:1 were calculated for the favipiravir peak using Open Lab CDS Software, respectively. That provided a 0.15 µg/mL level as a LOD and a 0.31 µg/mL level as a LOQ. Hence, we can say that the method is sensitive and can detect and quantify a very small concentration. The results are summarized in Table 4.

#### 3.2.7. Robustness

All the chromatographic variables that were deliberately altered (wavelength of detection, column oven temperature, composition of mobile phase, flow rate, and pH of the buffer) yielded findings that were well within the limit. Table 5 summarizes the results.

#### 3.2.8. Solution Stability

The analytical solution stability of the standard and sample was at least 24 h at room temperature. Table 6 provides a summary of the findings.

### 3.3. Isolation and Purification of Degradation Product

Favipiravir was significantly degraded in the presence of alkali. Hence, the degradation product from the alkali degradation solution was isolated by semipreparative HPLC using a YMC pack semipreparative HPLC column (250 mm × 10 mm, 5 µm particle size) with 0.1% formic acid in water and acetonitrile in a ratio of 85:15 *v/v* as a mobile phase at a flow rate of 5 mL/min at room temperature. The column eluent was monitored by a photodiode array detector at 322 nm. The collected degradation product was analyzed by HPLC using the conditions mentioned in Section 2.3.1. The isolated fractions had a purity greater than 98.5%, and the excess solvent was evaporated using a Rota evaporator (IKA, RV 10 auto pro V) followed by free drying (Labconco) to obtain the degradant in solid form. The collected degradant was characterized by UPLC ESI-TQ-MS/MS and ^1^H NMR.

### 3.4. Characterization of Alkali Degradation Products of Favipiravir Using UPLC-ESI-TQ-MS/MS and NMR

**Figure 5:**
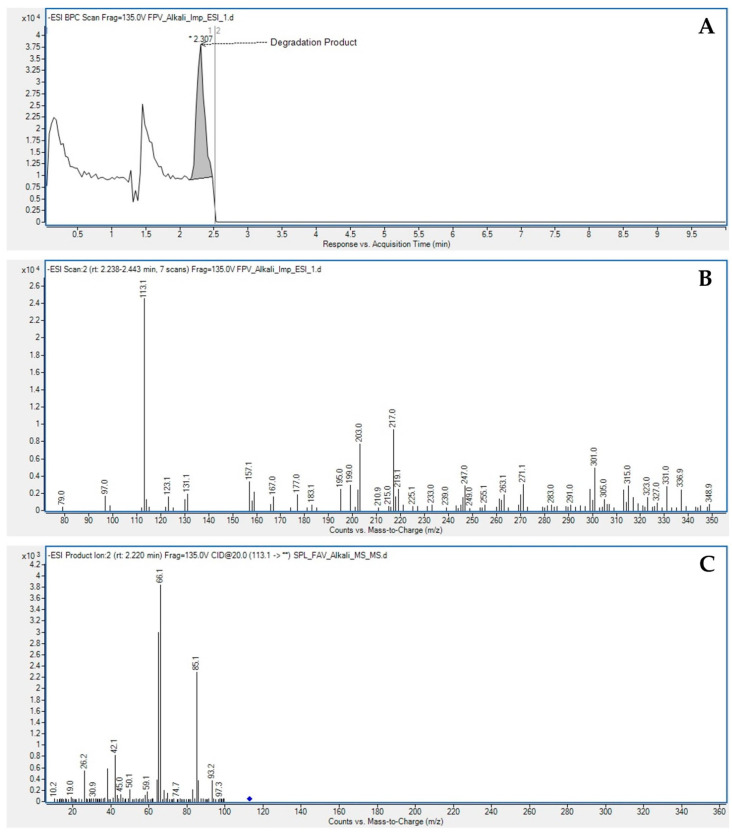
Base peak chromatogram (**A**); precursor ion scan (**B**); product ion scan (**C**) of alkali degradation sample using LC-MS/MS.

**Figure 6:**
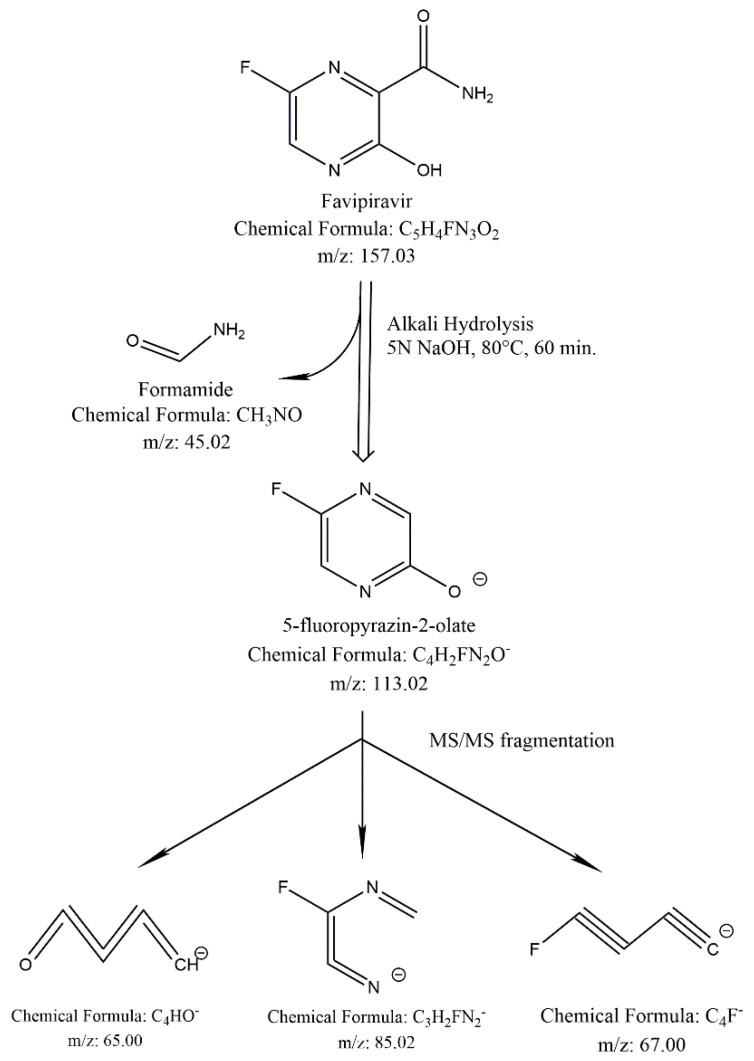
Plausible ESI-MS/MS fragmentation pattern of the alkali degradation product.

**Figure 7:**
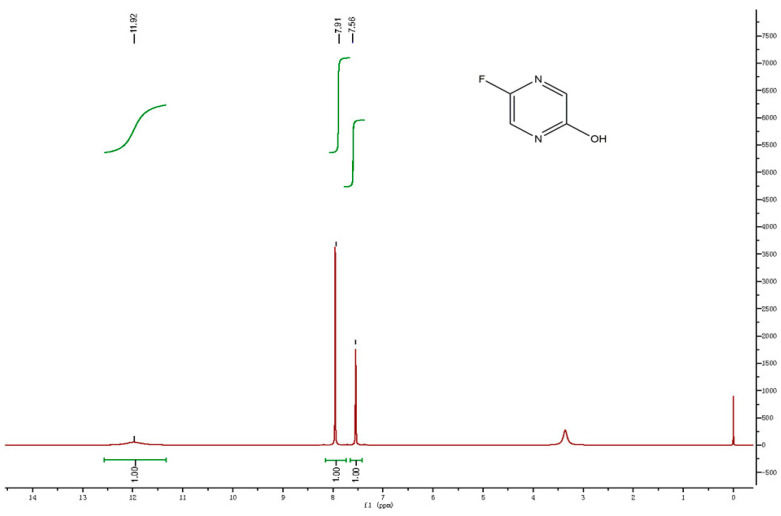
^1^H NMR of the isolated alkali degradation product.

In the LC-MS/MS spectral data, the mass of the alkali degradation product (DP-1) was observed as m/z 113.1 as [M-H]-, which was obtained by the elimination of the carboxamide group from favipiravir. The plausible fragmentations of DP-1 in negative mode under a collision energy (CE) of 20 V are shown in Figure 5. The alkali degradation product loses various functional groups, resulting in molecular ions with m/z values of 41.1, 66.1, and 85.1. A precursor ion scan in positive mode is shown in supplementary Figure S8. With the above information, the structure of the alkali degradation product (DP-1) could be characterized as 5-fluoropyrazin-2-ol. The plausible ESI-MS/MS fragmentation pattern of favipiravir and its alkali degradation product is summarized in Figure 6. The hydrogen proton signal at δ11.97 confirmed –OH (s, 1H); δ7.91 and δ7.56 confirmed –CH (s, 1H) in the proposed structure. The explanation is summarized in Figure 7.

## 4. Conclusions

The liquid chromatography method was optimized to separate favipiravir from its impurities and degradation products on a reversed-phase column in a favipiravir oral suspension. According to ICH recommendations, the forced degrading behavior of favipiravir in an oral suspension was investigated. Under stress conditions, it was observed that the prominent degradation product was generated under alkaline stress conditions. The potential degradation product was isolated, purified, and characterized using UPLC-ESI-TQ-MS/MS and ^1^H NMR. The degradation behavior indicates that the developed impurity may possess a risk of stability and can exceed the limits recommended by ICH Q3 during stability conditions. Method validation followed by identification along with the characterization of potential alkali degradation products were performed by UPLC-TQ-ESI-MS/MS and ^1^H NMR spectroscopy. The characterization data and degradation behavior under alkali conditions also derived a probable degradation path, which was discussed with scientific justification. The developed RP-HPLC method has been validated and is suitable for routine quantitative analysis of favipiravir in an oral suspension.

## Figures and Tables

**Table 1:** Summary data of forced degradation studies.

Mode of Degradation	Condition	Degradation Products	Rt (Min.)	Assay (%)	% Degradation	Peak Purity
Sample as such	No treatment	-	-	98.23	-	999.97
Acid	1 N HCl–1 mL/80 °C/60 min			97.42	-	999.95
	2 N HCl–1 mL/80 °C/60 min	-	-	97.35	-	999.96
	5 N HCl–1 mL/80 °C/60 min	-	-	96.25	1.98	999.90
Alkali	1 N NaOH–1 mL/80 °C/60 min	DP1	1.98	87.78	10.45	999.95
	2 N NaOH–1 mL/80 °C/60 min	DP1	1.98	81.76	16.47	999.92
	5 N NaOH–1 mL/80 °C/60 min	DP1	1.98	69.72	28.5	999.95
Oxidative	10% H_2_O_2_–1 mL/80 °C/60 min	-	-	97.90	-	999.96
	30% H_2_O_2_–1 mL/80 °C/60 min	DP1 DP2	1.98 6.48	94.73	3.50	999.95
Thermal	60 °C for 2 h	-	-	97.51	-	999.96
	80 °C for 1 h	-	-	97.86	-	999.95
Photolytic	UV light 254 nm	DP1 DP2	1.98 6.48	96.20	2.03	999.93

**Table 2:** Accuracy.

Recovery Levels	Amount Added Conc. (µg/mL)	Amount Recovered Conc. (µg/mL)	% Recovery
Level 1 (50%)	50.8	50.5	99.4
	50.2	49.8	99.2
	50.4	50.1	99.4
Level 2 (100%)	100.3	100.1	99.8
	100.6	99.98	99.3
	100.2	100.3	100.1
Level 3 (150%)	150.2	149.6	99.4
	150.8	150.2	99.6
	151.6	151.4	99.8
		Mean	99.55
		±SD	0.29
		%RSD	0.29

**Table 3:** System suitability and precision.

System Suitability		Intraday Precision (Set I)		Interday Precision (Set II)	
Injection No.	Area Count (mAU)	Sample No.	Assay (% Label Claim)	Sample No.	Assay (% Label Claim)
1	2416.372	1	97.63	1	97.96
2	2414.034	2	97.22	2	97.56
3	2428.586	3	96.60	3	97.16
4	2412.247	4	98.29	4	98.57
5	2411.316	5	98.78	5	98.78
		6	98.42	6	96.34
Mean	2416.511	Mean	97.66	Mean	97.73
±SD	7.02	±SD	0.78	±SD	0.91
%RSD	0.29	%RSD	0.79	%RSD	0.93

**Table 4:** Limit of detection and quantification.

Injection	5 (µg/mL)	2.5 (µg/mL)	1.25 (µg/mL)	0.625 (µg/mL)	0.31 (µg/mL)	0.15 (µg/mL)	0.07 (µg/mL)
	Area Count						
1	133.59	62.60	37.75	20.29	11.23	7.80	ND
2	132.42	65.49	34.55	20.97	11.26	7.51	ND
3	131.44	67.15	32.53	20.23	11.20	7.51	ND
Mean	132.48	65.15	34.94	20.50	11.23	7.61	-
±SD	1.07	2.30	2.63	0.41	0.03	0.17	-
%RSD	0.81	3.54	7.53	2.02	0.26	2.23	-

ND: Not detected.

**Table 5:** Robustness.

Method Parameters	Standard Solution			Assay (% Claim)
	Theoretical Plate	Tailing Factor	%RSD	
As such (Method precision)	3437.76	1.31	0.30	97.66
Variation in wavelength (320 nm)	3439.83	1.30	0.34	97.45
Variation in wavelength (324 nm)	3438.75	1.30	0.32	97.56
Variation in column oven temperature (20 °C)	3436.56	1.31	0.34	97.53
Variation in column oven temperature (30 °C)	3437.65	1.34	0.32	97.63
Variation in minor component in mobile phase (−5% of methanol)	3439.83	1.32	0.30	97.87
Variation in minor component in mobile phase (+5% of methanol)	3537.78	1.30	0.36	98.02
Variation in flow rate (0.8 mL/min)	3683.70	1.28	0.32	97.43
Variation in flow rate (1.2 mL/min)	3627.70	1.34	0.35	96.45
Variation in pH of Buffer solution (pH 4.3)	3438.78	1.35	0.33	97.56
Variation in pH of Buffer solution (pH 4.7)	3435.43	1.32	0.36	97.45

**Table 6:** Solution stability data.

Time (Hrs.)	Standard Solution		Sample Solution	
	Area Count	% Deviation	Area Count	% Deviation
Initial	2416.71	0.0	2370.65	0.0
4	2412.78	0.1	2360.90	0.4
10	2425.56	0.3	2345.92	1.0
15	2414.25	0.2	2386.86	0.6
24	2428.51	0.4	2365.03	0.2

## Data Availability

All data are available from R.P. upon request.

## Supplementary Materials

The following are available online at https://www.mdpi.com/article/10.3390/molecules27175606/s1. Figure S1: Acid Degradation Sample (1N HCl_1 mL_24 h); Figure S2: Alkali Degradation Sample (1N NaOH_5 mL_2 h); Figure S3: Peroxide Degradation Sample (10%H_2_O_2__1 mL_24 h); Figure S4: Peroxide Degradation Sample (30%H_2_O_2__1 mL_24 h); Figure S5: Thermal Degradation Sample (60 °C _2 h); Figure S6: Photolytic Degradation Sample (UV chamber at 254 nm, 6 h); Figure S7: UPLC-DAD chromatogram of alkali hydrolysis sample (Robustness purpose); Figure S8: MS/MS Product ion scan of alkali degradation product (Positive ion mode); Figure S9: Chromatogram of Favipiravir sample solution; Figure S10: Peak Purity spectra of favipiravir peak from sample solution.
