# Supplementary material for: Structural Elucidation of Alkali Degradation Impurities of Favipiravir from the Oral Suspension: UPLC-TQ-ESI-MS/MS and NMR
Source: Molecules. 2022 Aug 31;27(17):5606. doi: 10.3390/molecules27175606 (PMC9457609; doi:10.3390/molecules27175606)
Supplement: Supplementary file 1 [file molecules-27-05606-s001.zip › molecules-1872939-supplementary.pdf]

molecules

Article

Structure elucidation of alkali degradation impurity of Favipiravir from the oral suspension: UPLC-TQ-ESI-MS/MS and NMR

Ravi Patel[1,*], Abhishek Dube[1], Ravisinh Solanki[1], Dignesh Khunt[1], Shalin Parikh[2], Vijayabhaskarreddy Junnuthula[3,*], Sathish Dyawanapelly[4,*]

1 Graduate School of Pharmacy, Gujarat Technological University, Gandhinagar, 382028, Gujarat, India
2 Senores Pharmaceuticals Pvt. Ltd., Ahmedabad, 380058, Gujarat, India
3 Drug Research Program, Faculty of Pharmacy, University of Helsinki, Viikinkaari 5 E, 00790, Helsinki, Finland
4 Department of Pharmaceutical Sciences and Technology, Institute of Chemical Technology, Mumbai, 400019, India
* Correspondence: ravipatel.qa@gmail.com (R.P.); sa.dyawanapelly@ictmumbai.edu.in (S.D.); junnuthula.vijayabhaskarreddy@helsinki.fi (V.J.)

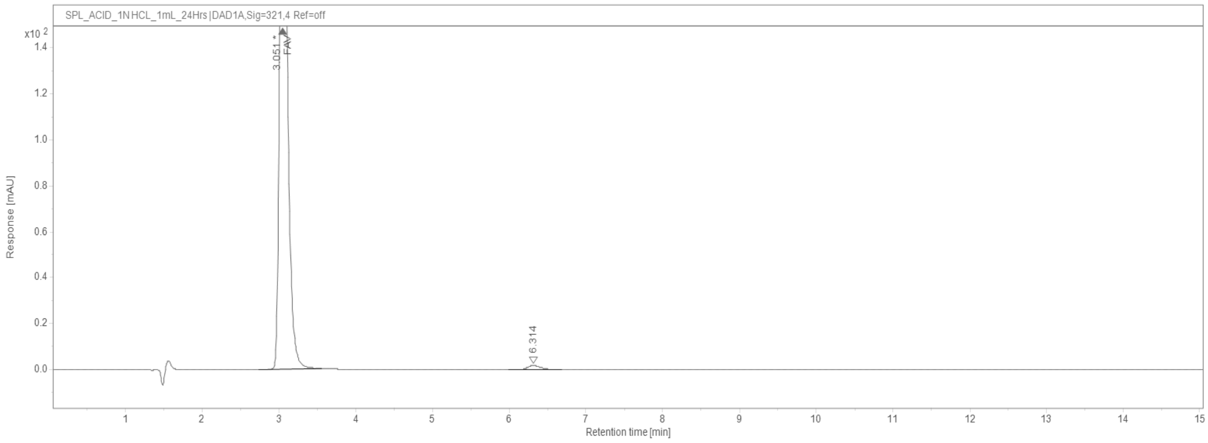

Figure S1: Acid Degradation Sample (1N HCl_1mL_24h)

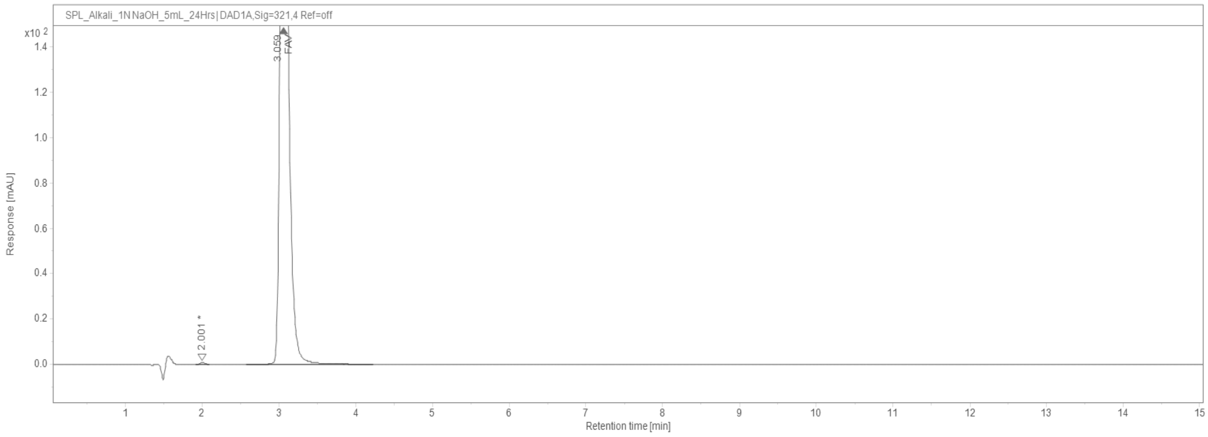

Figure S2: Alkali Degradation Sample (1N NaOH_5mL_24h)

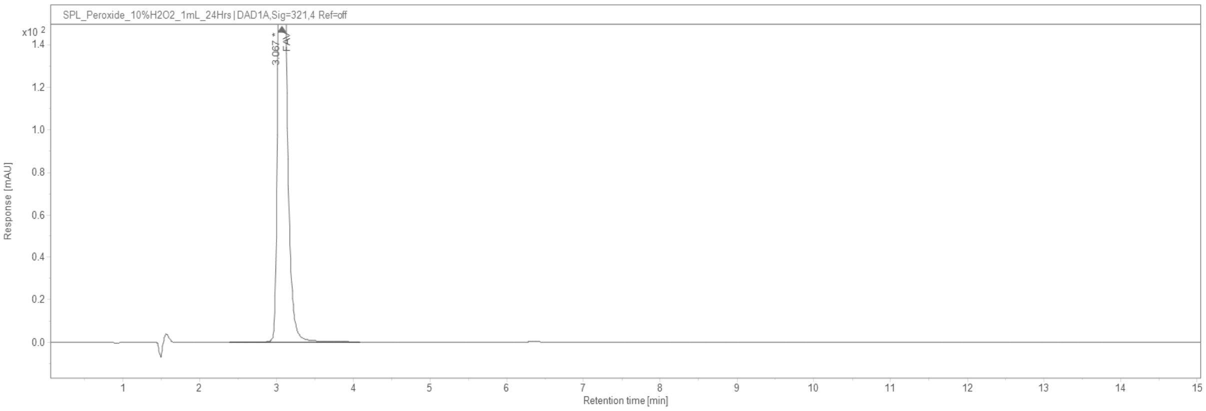

Figure S3: Peroxide Degradation Sample (10%H_2O_2_1mL_24h)

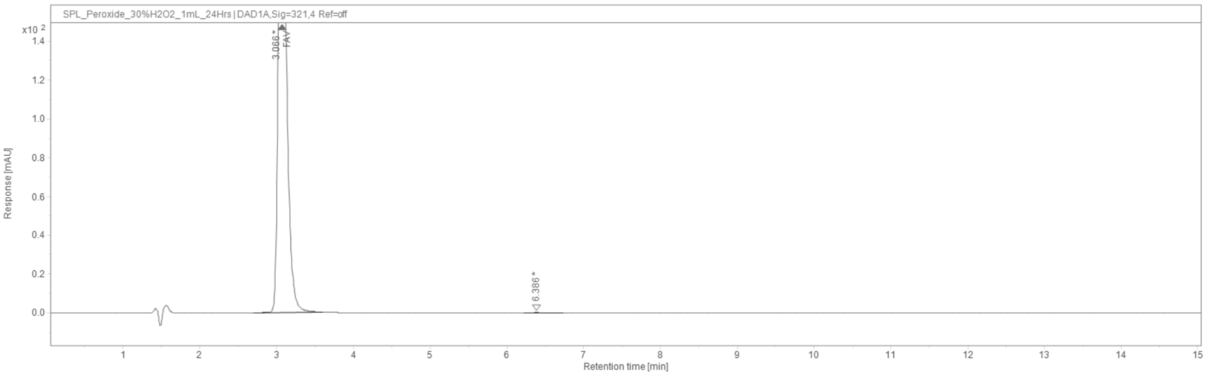

Figure S4: Peroxide Degradation Sample (30%H_2O_2_1mL_24h)

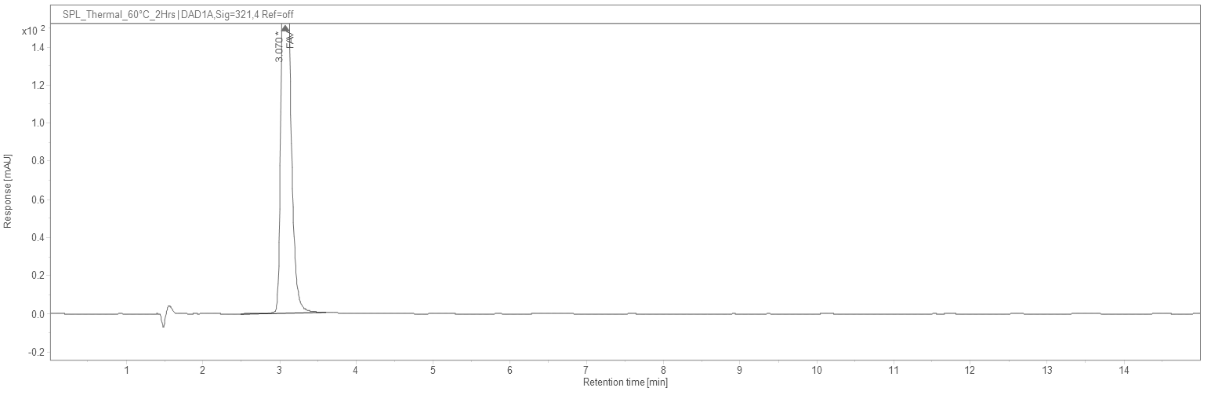

Figure S5: Thermal Degradation Sample (60°C _2h)

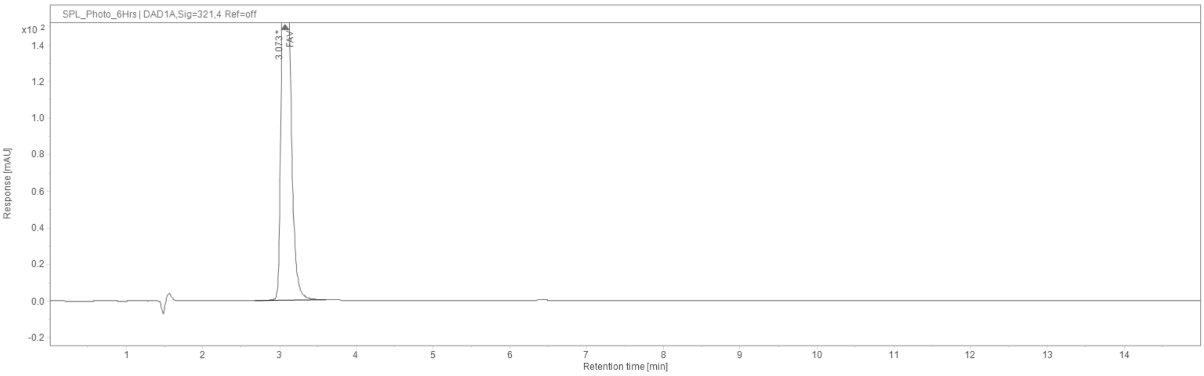

Figure S6: Photolytic Degradation Sample (UV chamber at 254nm, 6h)

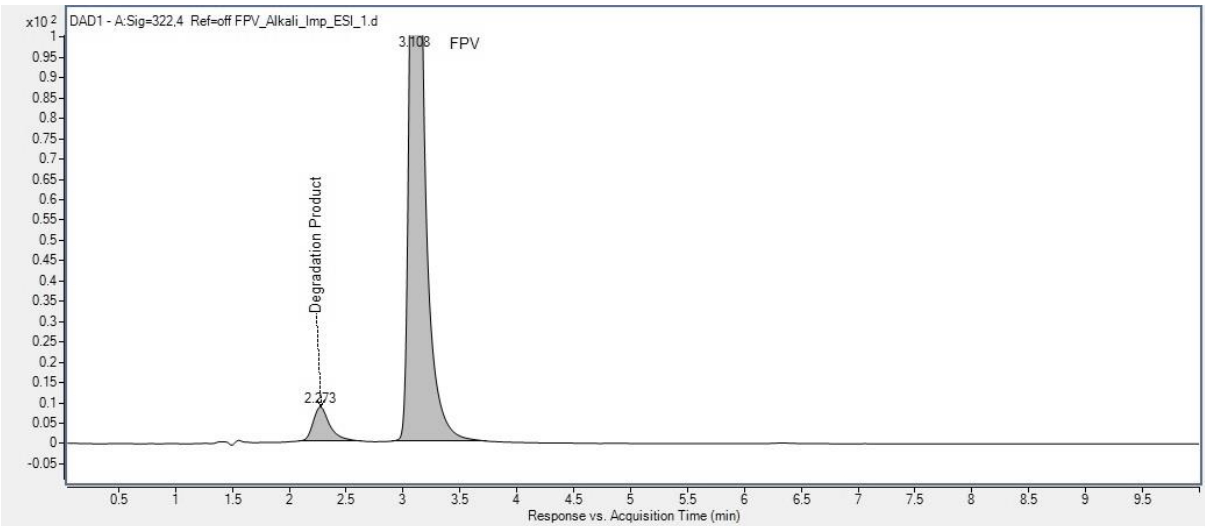

Figure S7: UPLC-DAD chromatogram of alkali hydrolysis sample (Intermediate Precision)

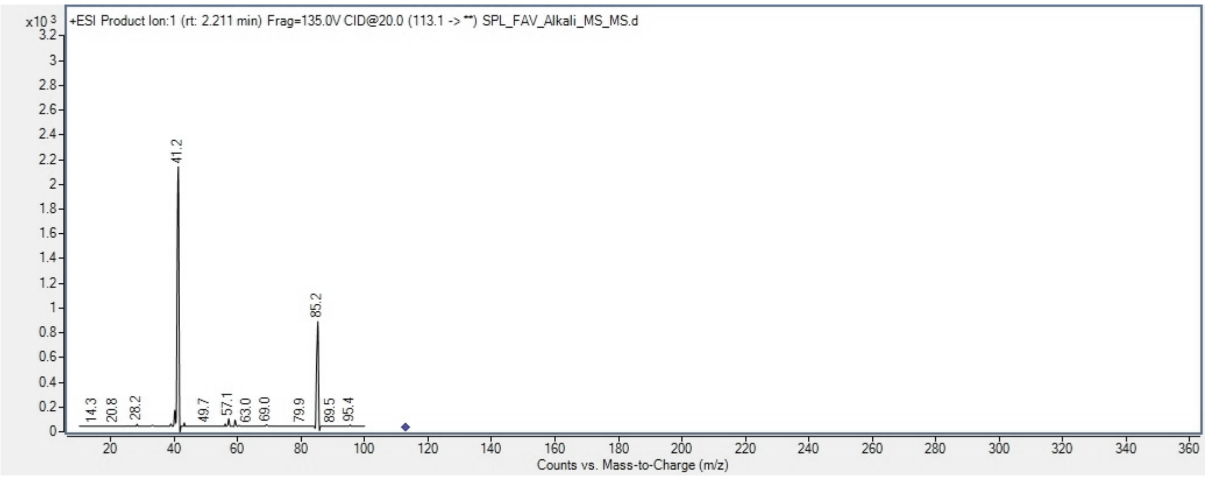

Figure S8: MS/MS Product ion scan of alkali degradation product (Positive ion mode)

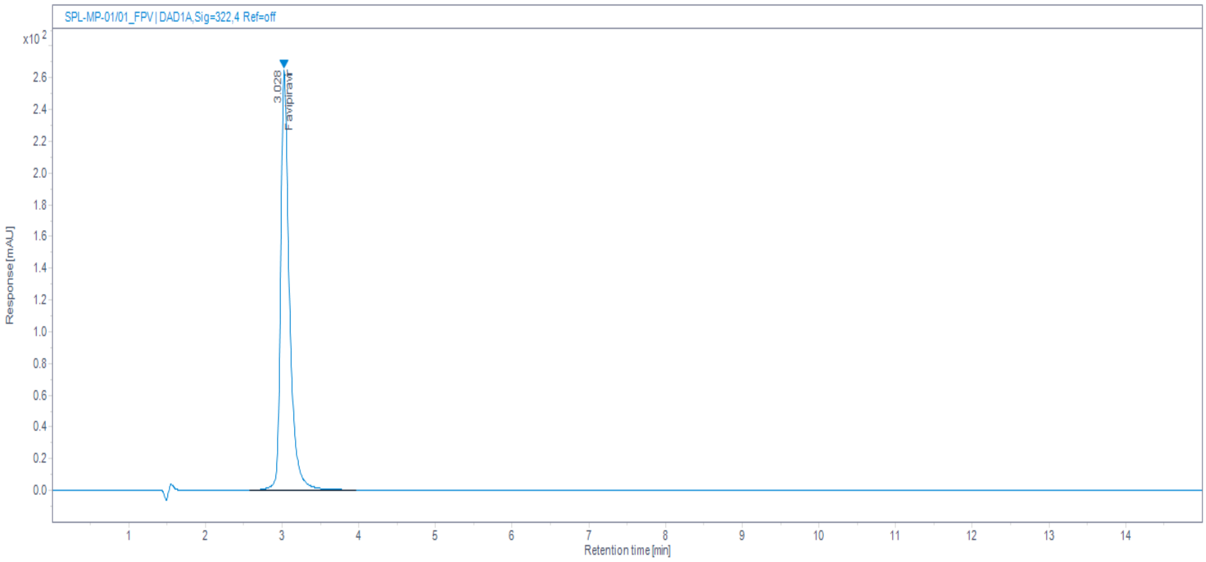

Figure S9: Chromatogram of Favipiravir sample solution

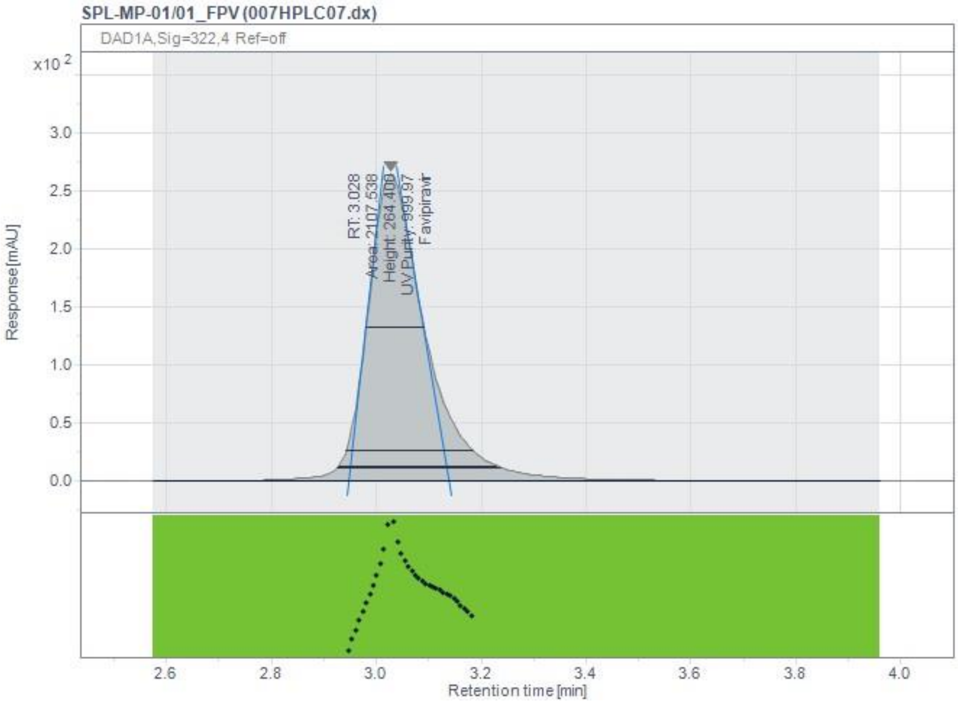

Figure S10: Peak Purity spectra of favipiravir peak from sample solution
